# Presenting symptoms of leprosy at diagnosis: Clinical evidence from a cross-sectional, population-based study

**DOI:** 10.1371/journal.pntd.0009913

**Published:** 2021-11-23

**Authors:** Xiaohua Chen, Shun Zha, Tie-Jun Shui

**Affiliations:** 1 Beijing Tropical Medicine Research Institute, Beijing Friendship Hospital, Capital Medical University, Beijing, China; 2 Beijing Key Laboratory for Research on Prevention and Treatment of Tropical Diseases, Capital Medical University, Beijing, China; 3 Yunnan Center for Disease Control and Prevention, Yunnan, China; Hospital Infantil de Mexico Federico Gomez, MEXICO

## Abstract

**Background:**

Leprosy is associated with different dermatologic and neurologic manifestations within a wide clinical spectrum, causing a great diagnostic challenge. Therefore, we aimed to examine associations between common presenting symptoms of leprosy and stage at diagnosis.

**Methodology/Principal findings:**

In this cross-sectional study, we analyzed population-level data from the Leprosy Management Information System (LEPMIS) in Yunnan, China, from 2010–2020 and enrolled patients with newly detected leprosy. The data of 2125 newly detected leprosy patients, with 5000 symptoms, were analyzed. Numbness (828/5000, 16.56%), erythema (802/5000, 16.04%), Painless nor pruritic skin lesions (651/5000, 13.02%), eyebrow hair loss (467/5000, 9.34%), and tubercles (442/5000, 8.84%) were common symptoms of leprosy. The symptoms related to skin (1935/2533, 76.39%) and leprosy reaction (279/297, 93.94%) were mainly existed in MB group. While the symptoms related to disability (263/316, 83.49%), clinical feature (38/56, 69.09%), and facial features (19/23, 82.61%) were predominantly presented in delayed diagnostic group. Despite low proportions, formic sensation (99/5000, 1.98%), pain (92/5000, 1.84%), pruritus (56/5000, 1.12%), finger contracture (109/5000, 2.18%), muscle atrophy (71/5000, 1.42%), and motor dysfunction (18/5000, 0.36%) were reported during the diagnosis of leprosy. The proportions of skin, skin and nerve, and nerve symptoms as initial symptoms were 33.25%, 44.95%, and 21.80% and as only symptoms were 28.66%, 57.81%, and 13.91%, respectively. In those with physical disability, nerve symptoms were the most frequent symptoms (57.65% and 65.36% for the initial and only symptoms, respectively) compared with skin and skin and nerve symptoms. In the delayed diagnosis group, nerve symptoms were the most frequent symptoms (15.73% and 17.25%) and were associated with the longest diagnostic intervals (mean±SD: 38.88±46.02 and 40.35±49.36 months for initial and only symptoms, respectively) when compared with skin and skin and nerve symptoms.

**Conclusions:**

Understanding the nature of presenting symptoms and developing symptom awareness campaigns would improve the level of leprosy awareness in the community. As nerve symptoms were related to a higher proportion of physical disability and longer diagnosis interval, we should increase awareness about nerve symptoms. Individuals with nerve symptoms should be considered the target group. Neurology outpatient visits may provide potential screening opportunities, and holding focused training for specialized neurology medical staff would enhance the capacity of the health system to recognize leprosy early.

## Author summary

Early diagnosis is key in leprosy control; however, the diagnosis of leprosy is still challenging. In this study, we reviewed the common and rare symptoms of leprosy. Numbness, erythema, painless nor pruritic skin lesions, eyebrow hair loss, and tubercles were common symptoms of leprosy. Despite low proportions, formic sensation, pain, pruritus, finger contracture, muscle atrophy, and motor dysfunction were also reported by leprosy patients. As initial symptoms at symptom onset and only symptoms at diagnosis, nerve symptoms were associated with a greater degree of physical disability and a longer diagnosis interval than skin and skin and nerve symptoms. Understanding the presenting symptoms in detail would improve the level of leprosy awareness in the community. Increasing awareness of nerve symptoms, focusing on individuals with nerve symptoms at neurology outpatient visits, and holding focused training for medical staff specializing in neurology would enhance the capacity of the health system to recognize leprosy early.

## Introduction

Leprosy is a chronic granulomatous infectious disease caused by the bacterium *Mycobacterium leprae (M*. *leprae)*, an intracytoplasmic parasite of macrophages and Schwann cells. Depending on the immunologic status of the host, the clinical picture can range from localized to disseminated and self-limiting to progressive. The disease primarily affects the superficial peripheral nervous system and the skin, but it may also involve the upper respiratory tract mucosa, anterior chambers of the eyes, bones, and testes [1].

Although the prevalence and incidence rates for leprosy have been significantly reduced as a result of the control strategies of the World Health Organization (WHO), new cases still occur [1]. Every year, >200,000 new leprosy cases are registered globally. This number has been fairly stable over the past several years [2]. A total of 202,185 new cases were reported from 160 countries in 2019, corresponding to the global new-case detection rate of 25.9 per one million population, with 10,813 leprosy cases associated with grade 2 disability (G2D) at diagnosis globally. The proportion of G2D cases among new cases was 5.3%, corresponding to 1.7 per one million population.

Early diagnosis and prompt treatment of all new cases of leprosy with multidrug therapy (MDT) remain the key strategies for leprosy control [3]. However, as one of the great imitators, the disease exhibits different dermatologic and neurologic manifestations within a wide clinical spectrum, which causes a great diagnostic challenge [1]. Delayed diagnosis of leprosy has been reported globally [4–6].

As early diagnosis minimizes damage and disability, early symptoms need to be recognized to prevent long-term sequelae associated with irreversible nerve damage [6]. However, the presenting symptoms of leprosy, especially those associated with different diagnostic intervals, have not been described in detail. In this study, we therefore aimed to examine associations between common presenting symptoms of leprosy, different classifications of leprosy, and different diagnostic intervals using data from a population-based cohort of patients with incident leprosy.

## Methods

### Ethics statement

Ethical approval for this study was obtained by the ethics committee of the Yunnan CDC, Yunnan, China. The data extracted from LEPMIS, which is anonymous without individually identifying data. Individual identifying information was not available and therefore not used.

### Study design

For this cross-sectional, population-based study, we analyzed data of patients included in the Leprosy Management Information System (LEPMIS) in Yunnan, China, from January 1, 2010, to December 31, 2020. Trained staff and/or experienced clinicians enter basic information about the disease and the medical data of patients into the database. The data were collated by Yunnan Center for Disease Control and Prevention (CDC).

### Participants

Yunnan, China, bears a significant leprosy burden. The characteristics and types of included patients were representative of a leprosy cohort in contemporary China. Our study was restricted by available data for newly detected leprosy cases, including patients’ basic demographic information (sex, date of birth, and ethnicity) and clinical information (age at confirmed diagnosis, date of symptom onset, date of confirmed diagnosis, duration from symptom onset to confirmed diagnosis, chief complaint, leprosy reaction, grade of physical impairment, Ridley–Jopling classification, and WHO operational classification).

Newly detected leprosy cases were diagnosed by medical staff specializing in leprosy and verified by the Yunnan Province CDC. Leprosy diagnosis was established based on clinical signs and symptoms, skin smears, skin biopsies, and neurophysiologic examinations. The leprosy patients were classified into the following groups based on the Ridley–Jopling [7] classification: tuberculoid (TT), borderline-tuberculoid (BT), borderline-borderline (BB), borderline-lepromatous (BL), and lepromatous (LL) groups and indeterminate (I). Leprosy patients were also classified as multibacillary (MB) or paucibacillary (PB) according to the WHO operational classification [8].

### Procedures

For data analysis in this study, leprosy patients were divided into MB and PB groups. The study population was stratified by diagnostic intervals: early diagnosis and delayed diagnosis. The term “early detection” was used if the time between disease onset and diagnosis was within 2 years and the patient had grade 0 or grade 1 disability according to the WHO definition of leprosy-associated disability [8]. “Delayed diagnosis” was defined as a duration between disease onset and diagnosis of more than 2 years and/or grade 2 disability according to the WHO definition of leprosy-associated disability [8]. Information about presenting symptoms was extracted from chief complaint data. In terms of initial symptoms and only symptoms, the symptoms were divided into “skin symptoms”, “skin and nerve symptoms”, and “nerve symptoms”.

### Statistical analysis

Statistical analysis involved comparisons of demographic data and symptoms among groups. The chi-square and Fisher’s exact tests were utilized to compare distributions of categorical variables between groups. The t test was used for continuous variables. Statistical analysis was performed using GraphPad Prism software version 5.0 (GraphPad Software Inc., San Diego, CA, USA), and statistical significance was assessed at the 0.05 level.

## Results

### Characteristics of newly detected leprosy patients

**Fig 1:**
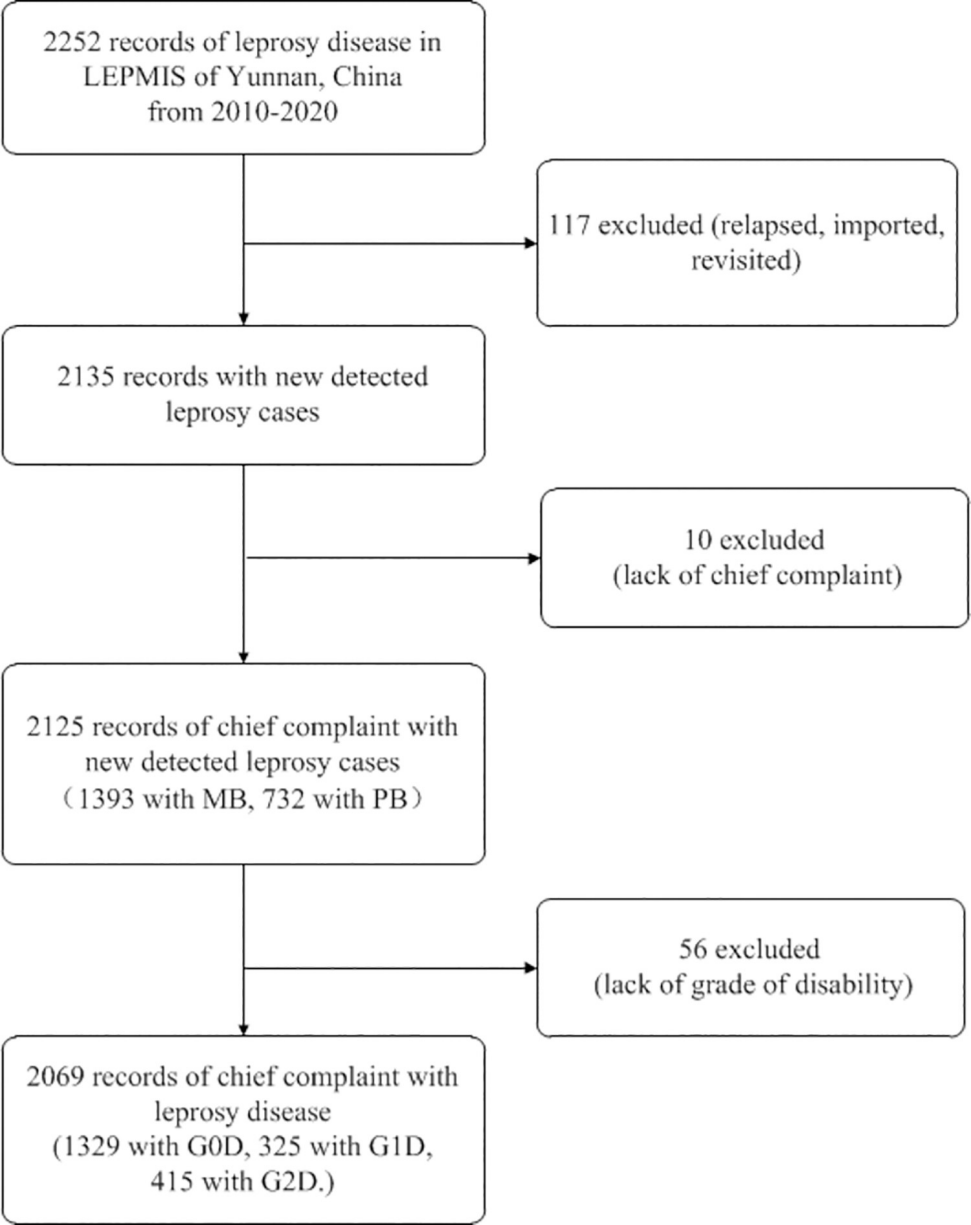
Study Population.

During the eleven-year study period from 2010 to 2020, 2252 records of leprosy cases in Yunnan, China, were retrieved; 117 records were excluded because they were relapsed, imported, or revisited cases, and 10 records were excluded because they were missing chief complaint information (Fig 1). As early diagnosis and delayed diagnosis are related to the grade of physical disability due to leprosy, 56 records were excluded due to a lack of a physical disability grade in the early and delayed diagnosis analysis.

A total of 2125 new cases of leprosy were identified; 698 (32.85%) occurred in females, 1174 (55.25%) occurred in patients belonging to a ethnic minority, 1771 (83.34%) occurred in people between 15 and 59 years of age, 1830 (86.12%) occurred in farmers, 1534 (72.19%) occurred in married individuals, and 1969 (92.66%) occurred in those with an incomplete high school education (Table 1).

**Table 1:** Basic Characteristics of the Newly Detected Leprosy Cases Enrolled in This Study in Yunnan, China from 2010–2020.

		Leprosy	(n,%)	MB	(n,%)	PB	(n,%)	P value (MB *vs* PB)	Early diagnosis	(n,%)	Delayed diagnosis	(n,%)	P value (Early *vs* Delayed)
	Total	2125	100,00%	1393	65,55%	732	34,45%		1249	58,78%	820	38,59%	
Gender	Male	1427	67,15%	944	67,77%	483	65,98%	0,405	819	65,57%	567	69,15%	0,0945
	Female	698	32,85%	449	32,23%	249	34,02%		430	34,43%	253	30,85%	
Age	Median (IQR), y	40	(29–52)	39	(29–50.5)	42	(29–56)	0.0079*	38	(27–49)	44	(32–56)	<0.0001*
	Mean(Min-Max)	41.13±16.07	(4–89)	40.43±15.18	(9–89)	42.37±17.49	(4–88)		38.76±15.45	(4–89)	44.47±16.19	(9–88)	
Age group	<15 years	60	2,82%	23	1,65%	37	5,05%	<0.0001*	50	4,00%	8	0,98%	<0.0001*
	15–19 years	125	5,88%	82	5,89%	43	5,87%		83	6,65%	40	4,88%	
	20–29 years	374	17,60%	268	19,24%	106	14,48%		248	19,86%	117	14,27%	
	30–39 years	472	22,21%	337	24,19%	135	18,44%		295	23,62%	166	20,24%	
	40–49 years	454	21,36%	309	22,18%	145	19,81%		266	21,30%	176	21,46%	
	50–59 years	346	16,28%	209	15,00%	137	18,72%		176	14,09%	163	19,88%	
	60–69 years	192	9,04%	106	7,61%	86	11,75%		93	7,45%	90	10,98%	
	70–79 years	89	4,19%	53	3,80%	36	4,92%		34	2,72%	51	6,22%	
	≥80 years	13	0,61%	6	0,43%	7	0,96%		4	0,32%	9	1,10%	
Ethnics	Han	951	44,75%	662	47,52%	289	39,48%	<0.0001*	512	40,99%	418	50,98%	<0.0001*
	Hui	8	0,38%	3	0,22%	5	0,68%		3	0,24%	5	0,61%	
	Tibetan	19	0,89%	15	1,08%	4	0,55%		9	0,72%	10	1,22%	
	Miao	307	14,45%	127	9,12%	180	24,59%		219	17,53%	84	10,24%	
	Yi	289	13,60%	176	12,63%	113	15,44%		174	13,93%	108	13,17%	
	Zhuang	164	7,72%	100	7,18%	64	8,74%		109	8,73%	50	6,10%	
	Man	1	0,05%	1	0,07%	0	0,00%		1	0,08%	0	0,00%	
	Yao	16	0,75%	12	0,86%	4	0,55%		12	0,96%	4	0,49%	
	Bai	44	2,07%	28	2,01%	16	2,19%		22	1,76%	22	2,68%	
	Hani	44	2,07%	37	2,66%	7	0,96%		29	2,32%	13	1,59%	
	Dai	113	5,32%	75	5,38%	38	5,19%		75	6,00%	34	4,15%	
	Lisu	45	2,12%	32	2,30%	13	1,78%		45	3,60%	24	2,93%	
	Wa	9	0,42%	7	0,50%	2	0,27%		2	0,16%	6	0,73%	
	Lahu	93	4,38%	66	4,74%	27	3,69%		46	3,68%	35	4,27%	
	Naxi	7	0,33%	6	0,43%	1	0,14%		5	0,40%	2	0,24%	
	Jingpo	6	0,24%	5	0,36%	1	0,14%		4	0,32%	2	0,24%	
	Bulang	2	0,09%	2	0,14%	0	0,00%		1	0,08%	1	0,12%	
	Achang	1	0,05%	1	0,07%	0	0,00%		1	0,08%	0	0,00%	
	Pumi	1	0,05%	1	0,07%	0	0,00%		0	0,00%	0	0,00%	
	De’Ang	1	0,05%	1	0,07%	0	0,00%		1	0,08%	0	0,00%	
	Jinuo	2	0,09%	0	0,00%	2	0,27%		2	0,16%	0	0,00%	
	Other	2	0,09%	2	0,14%	0	0,00%		1	0,08%	0	0,00%	
Occupation	Nursery child	2	0,09%	0	0,00%	2	0,27%	0.0146*	1	0,08%	0	0,00%	0.0085*
	Children	2	0,09%	1	0,07%	1	0,14%		2	0,16%	0	0,00%	
	Student	115	5,41%	62	4,45%	53	7,24%		90	7,21%	24	2,93%	
	Teacher	9	0,42%	7	0,50%	2	0,27%		5	0,40%	4	0,49%	
	Food and beverage industry	1	0,05%	1	0,07%	0	0,00%		1	0,08%	0	0,00%	
	Public place attendant	2	0,09%	0	0,00%	2	0,27%		2	0,16%	0	0,00%	
	Business Services	5	0,24%	3	0,22%	2	0,27%		5	0,40%	0	0,00%	
	Medical staff	1	0,05%	1	0,07%	0	0,00%		1	0,08%	0	0,00%	
	Worker	26	1,22%	18	1,29%	8	1,09%		14	1,12%	12	1,46%	
	Farmer labourer	42	1,98%	34	2,44%	8	1,09%		24	1,92%	36	4,39%	
	Farmer	1830	86,12%	1202	86,29%	628	85,79%		1054	84,39%	705	85,98%	
	Herdsman	2	0,09%	0	0,00%	2	0,27%		2	0,16%	0	0,00%	
	Seafarers and long-distance drivers	3	0,14%	3	0,22%	0	0,00%		1	0,08%	2	0,24%	
	Clerks	6	0,28%	4	0,29%	2	0,27%		4	0,32%	2	0,24%	
	Retired	16	0,75%	11	0,79%	5	0,68%		10	0,80%	6	0,73%	
	Housework and unemployment	33	1,55%	23	1,65%	10	1,37%		17	1,36%	16	1,95%	
	others	15	0,71%	9	0,65%	6	0,82%		5	0,40%	9	1,10%	
	Technical personnel	1	0,05%	1	0,07%	0	0,00%		1	0,08%	0	0,00%	
	Self employed	12	0,56%	11	0,79%	1	0,14%		8	0,64%	4	0,49%	
	Unknown	2	0,09%	0	0,00%	2	0,27%		2	0,16%	0	0,00%	
Education	College education	49	2,31%	36	2,58%	13	1,78%	*0*,*0519*	30	2,40%	19	2,32%	0,1463
	Senior high school	81	3,81%	62	4,45%	19	2,60%		53	4,24%	28	3,41%	
	Junior high school	629	29,60%	425	30,51%	204	27,87%		392	31,39%	220	26,83%	
	Primary school	876	41,22%	569	40,85%	307	41,94%		509	40,75%	346	42,20%	
	Illiteracy	460	21,65	282	20,24%	178	24,32%		248	19,86%	195	23,78%	
	Preschool	4	0,19%	16	1,15%	4	0,55%		3	0,24%	0	0,00%	
	Missing data	26	1,32%	19	1,36%	10	1,37%		14	1,12%	12	1,46%	
Marriage	Unmarried	453	21,32%	301	21,61%	152	20,77%	*0*,*836*	300	24,02%	142	17,32%	
	Married	1534	72,19%	1003	72,00%	531	72,54%		881	70,54%	612	74,63%	
	Widowed	75	3,53%	49	3,52%	26	3,55%		34	2,72%	38	4,63%	
	Divorce	46	2,16%	31	2,23%	15	2,05%		24	1,92%	21	2,56%	
	Unknown	17	0,80%	9	0,65%	8	1,09%		10	0,80%	7	0,85%	
Leprosy classification													
Ridley and Jopling	I	28	1,32%	0	0,00%	28	3,83%	<0.0001*	13	1,04%	14	1,71%	<0.0001*
	TT	202	9,46%	0	0,00%	201	27,46%		95	7,61%	104	12,68%	
	BT	503	23,67%	0	0,00%	503	68,72%		282	22,58%	215	26,22%	
	BB	197	9,27%	197	14,14%	0	0,00%		131	10,49%	62	7,56%	
	BL	912	42,92%	912	65,47%	0	0,00%		570	45,64%	311	37,93%	
	LL	283	13,36%	284	20,39%	0	0,00%		158	12,65%	114	13,90%	
WHO**	PB	732	34,45%	0	0,00%	732	100,00%	<0.0001*	390	31,22%	333	40,61%	<0.0001*
	MB	1393	65,55%	1393	100,00%	0	0,00%		859	68,78%	487	59,39%	
Leprosy reactions	Total	285	13,41%	225	16,15%	60	8,20%	<0.0001*	176	14,09%	86	10,49%	
	T1R	121	5,69%	71	5,10%	51	6,97%		80	6,41%	37	4,51%	
	T2R	122	5,74%	115	8,26%	6	0,82%		66	5,28%	46	5,61%	
	Mixed	42	1,98%	39	2,80%	3	0,41%		30	2,40%	8	0,98%	
Physical disability	Grade 0	1329	62,54%	926	66,48%	403	55,05%	<0.0001*	1040	83,27%	289	35,24%	<0.0001*
	Grade 1	325	15,29%	230	16,51%	95	12,98%		209	16,73%	116	14,15%	
	Grade 2	415	19,53%	190	13,64%	225	30,74%		0	0,00%	415	50,61%	
	Missing data	56	2,64%	47	3,37%	9	1,23%		0	0,00%	0	0,00%	
Diagnosis intervals	Early Diagnosis	1249	58,78%	859	61,67%	390	53,28%	<0.0001*	1249	100,00%	0	0,00%	<0.0001*
	Delayed Diagnosis	820	38,59%	487	34,96%	333	45,49%		0	0,00%	820	100,00%	
	Missing data	56	2,64%	47	3,37%	9	1,23%		0	0,00%	0	0,00%	
Duration from Symptom onset to clinical confirmation,	Median (IQR)	20	(29–52)	21	(17–26)	20	(17–30)	*0*,*0611*	18	(16–21)	34	(25–56)	<0.0001*
	Mean±SD (Min-Max)	29.54±33.57	(1–364)	*28*.*56±31*.*7*	(1–361)	*31*.*43±36*.*83*	(1–364)		*17*.*29±5*.*135*	(1–23)	*48*.*68±47*.*46*	(1–364)	

I: inderminate;TT: tuberculoid (TT); BT: borderline-tuberculoid; BB: borderline-borderline;BL: borderline-lepromatous;LL:lepromatous.MB:multibacillary; PB: paucibacillary. WHO

**: world health orgnazation.

*P<0.05.

T1R:Reversal reaction or type 1 reaction, T2R:Erythema nodosum leprosum or type 2 reaction.

Regarding operational classification, 1393 cases (65.55%) were MB, and 732 (34.45%) were PB (Table 1). Regarding Ridley–Jopling classification, the predominant form was BL (n = 912, 42.92%), followed by BT (n = 503, 23.67%), LL (n = 284, 13.36%), TT (n = 201, 9.46%), BB (n = 197, 9.27%), and indeterminate (I) (n = 28, 1.32%) (Table 1). A leprosy reaction was reported in 285 (13.41%) cases (Table 1). With regard to physical disability, 1239 (58.78%) patients had grade 0 disability, 325 (15.29%) had grade 1 disability, and 415 (19.53%) had grade 2 disability (Table 1).

### Symptom signatures of leprosy

Among 2125 newly detected cases of leprosy disease with symptom signatures, 5000 symptoms were recorded, averaging 2.4 symptoms per case. A total of 34 distinct presenting symptoms were reported in the study population, and 76 symptoms were described in LEPMIS (Table 2). The presenting symptoms were divided into symptoms related to clinical features, skin (skin appendages and skin lesions), nerves, leprosy reactions, disability, and facial and other organ features (Table 2). The predominant symptoms were skin symptoms (n = 2533, 50.66%), followed by nerve symptoms (n = 1775, 35.50%), disability (n = 316, 6.32%), and leprosy reactions (n = 297, 5.94%) (Table 3). Symptoms related to skin (1935/2533, 76.39%) and leprosy reactions (279/297, 93.94%) mainly occurred in the MB group. Symptoms related to disability (263/316, 83.23%), clinical features (38/56, 69.09%), and facial features (19/23, 82.61%) predominantly occurred in the delayed diagnosis group (Table 3).

**Table 2:** The defined symptoms of interest as derived from those described in the LEPMIS in Yunnan, China 2010–2020.

Symptom construct	Symptom(s) as originally described in LEPMIS	Category
Injury	Injury	Clnical feature
Refractory	Refractory	Clnical feature
Weak	Weak	Clnical feature
Eyebrows hair loss	Eyebrows fall off	Skin Symptoms (Skin appendages)
	Eyelashes loss	Skin Symptoms (Skin appendages)
	Hair Loss	Skin Symptoms (Skin appendages)
Anhidrosis & Dryness	Anhidrosis	Skin Symptoms (Skin appendages)
	Hypohidrosis	Skin Symptoms (Skin appendages)
	Dryness	Skin Symptoms (Skin appendages)
Infiltration	Facial infiltration	Skin Symptoms (Skin lesion)
	Auricular infiltration	Skin Symptoms (Skin lesion)
	Trunk infiltration	Skin Symptoms (Skin lesion)
Erythema	Erythema	Skin Symptoms (Skin lesion)
	Erythema Pallidum	Skin Symptoms (Skin lesion)
	Dark Erythema	Skin Symptoms (Skin lesion)
Tubercle	Tubercle	Skin Symptoms (Skin lesion)
Blisters	Blisters	Skin Symptoms (Skin lesion)
Light-colored spot	Light-colored spot	Skin Symptoms (Skin lesion)
	White spot	Skin Symptoms (Skin lesion)
	Pale white spot	Skin Symptoms (Skin lesion)
Plaque	Plaque	Skin Symptoms (Skin lesion)
	Papules	Skin Symptoms (Skin lesion)
	Rashes	Skin Symptoms (Skin lesion)
	Skin lesion	Skin Symptoms (Skin lesion)
	Mass	Skin Symptoms (Skin lesion)
Brown spot	Brown spot	Skin Symptoms (Skin lesion)
	Black spot	Skin Symptoms (Skin lesion)
Moss	Scale	Skin Symptoms (Skin lesion)
	Moss	Skin Symptoms (Skin lesion)
Numbness	Numbness	Nerve symptoms
Painless nor pruritic skin lesions	Neither pain nor pruritus of skin lesion	Nerve symptoms
Pain	Pain	Nerve symptoms
Formic sensation	Formic sensation	Nerve symptoms
	Creeping sensation	Nerve symptoms
	Needling sensation	Nerve symptoms
	Burning sensation	Nerve symptoms
Pruritus	Pruritus	Nerve symptoms
Sensory disturbance	Sensory disturbance	Nerve symptoms
	Loss of sensation	Nerve symptoms
	Insensibility to pain	Nerve symptoms
	Hypoesthesia	Nerve symptoms
	Paresthesia	Nerve symptoms
Redness and/or swelling	Facial redness	Leprosy reaction (I/II)
	Facial redness and swelling	Leprosy reaction (I/II)
	Facial Edema	Leprosy reaction (I/II)
	Limbs Edema	Leprosy reaction (I/II)
	Limbs redness and swelling	Leprosy reaction (I/II)
	Limbs redness	Leprosy reaction (I/II)
	Body redness and swelling	Leprosy reaction (I/II)
	Body Edema	Leprosy reaction (I/II)
Fever	Fever	Leprosy reaction (I/II)
	High fever	Leprosy reaction (I/II)
Erythema nodosum	Erythema nodosum	Leprosy reaction (I/II)
Ulcer	Ulcer	Disability
	Fester and erode	Disability
	Ulceration	Disability
	Chapped	Disability
Finger contracture	Hook fingers	Disability
Muscle atrophy*	Muscle Atrophy	Disability
Shortening of fingers and toes	Shortening of fingers and toes	Disability
Motor dysfunction	Motor dysfunction	Disability
	Stiff	Disability
Claw hand and/or foot	Claw hand	Disability
	Claw foot	Disability
Eyelids Incomplete closure	Eyelids Incomplete closure	Facial feature
Red eyes	Red eyes	Facial feature
Angle of mouth askew /facial paralysis	Angle of mouth askew	Facial feature
	Facial paralysis	Facial feature
Nasal symptoms	Nose collapsed	Facial feature
	Nasal deformity	Facial feature
	Nasal Erythema	Facial feature
	Nasal numbness	Facial feature
	Nasal congestion	Facial feature
	Turbinate enlargement	Facial feature
Larynx	Hoarseness	Other organ
Scrotum	Scrotal swelling	Other organ

* Muscle atrophy presented in hand and lower limbs.

**Table 3:** Observed Proportions of Presenting Symptoms in Newly Detected Leprosy Cases in Yunnan, China, 2010–2020.

	Leprosy				MB				PB				P value	Early diagnosis				Delayed diagnosis				P value
	N	%	95%CI		N	%	95%CI		N	%	95%CI			N	%	95%CI		N	%	95%CI		
Clinical features	56	1,12%	0,83%	1,41%	27	0,78%	0,49%	1,08%	29	1,84%	1,18%	2,51%	0,8502	17	0,59%	0,31%	0,87%	38	1,93%	1,32%	2,53%	0.0001*
Skin	2533	50,66%	49,27%	52,05%	1935	56,22%	54,56%	57,87%	612	38,93%	36,52%	41,34%	<0.0001*	1581	54,91%	53,10%	56,73%	865	43,86%	41,67%	46,05%	<0.0001*
(Skin appendages)	656	13,12%	12,18%	14,06%	546	15,86%	14,64%	17,08%	110	7,00%	5,74%	8,26%	<0.0001*	364	12,64%	11,43%	13,86%	262	13,29%	11,79%	14,78%	<0.0001*
(Skin lesion)	1877	37,54%	36,20%	38,88%	1389	40,35%	38,72%	41,99%	502	31,93%	29,63%	34,24%	<0.0001*	1217	42,27%	40,47%	44,08%	603	30,58%	28,54%	32,61%	<0.0001*
Nerve	1775	35,50%	34,17%	36,83%	1080	31,38%	29,83%	32,93%	695	44,21%	41,76%	46,67%	<0.0001*	1050	36,47%	34,71%	38,23%	674	34,18%	32,09%	36,27%	<0.0001*
Leprosy reaction (I/II)	297	5,94%	5,28%	6,60%	279	8,11%	7,19%	9,02%	18	1,15%	0,62%	1,67%	<0.0001*	175	6,08%	5,21%	6,95%	113	5,73%	4,70%	6,76%	<0.0001*
Disability	316	6,32%	5,65%	6,99%	104	3,02%	2,45%	3,59%	212	13,49%	11,80%	15,17%	<0.0001*	52	1,81%	1,32%	2,29%	263	13,34%	11,84%	14,84%	<0.0001*
Facial feature & Other organ	23	0,46%	0,27%	0,65%	17	0,49%	0,26%	0,73%	6	0,38%	0,08%	0,69%	0.0028*	4	0,14%	0,00%	0,28%	19	0,96%	0,53%	1,39%	<0.0001*

MB: multibacillary, PB: paucibacillary.

*P<0.05.

Skin symptoms were divided into skin lesions (n = 1877, 37.54%) and skin appendage symptoms (656, 13.12%). The common symptoms of skin lesions were erythema (n = 802, 16.04%), tubercles (n = 442, 8.84%), plaques (n = 231, 4.62%), infiltration (139, 2.78%), light-colored spots (n = 129, 2.58%), and blisters (n = 107, 2.14%). The common symptoms affecting skin appendages were eyebrow hair loss (n = 467, 9.34%) and anhidrosis and dryness (n = 189, 3.78%). Regarding nerve symptoms, the most common symptom was numbness (n = 828, 16.56%), followed by painless nor pruritic skin lesions (n = 651, 13.02%). It is worth noting that formic sensation (n = 99, 1.98%), pain (n = 92, 1.84%), and pruritus (n = 56, 1.12%) were also reported by patients with leprosy. Redness and/or swelling was the most common symptom of a leprosy reaction (n = 257, 5.14%). Finger contracture (n = 109, 2.18%), ulcers (n = 101, 2.02%), and muscle atrophy (n = 71, 1.42%) were three most common symptoms of physical disability (Table 3). Injury (n = 14, 0.30%), refractory illness (n = 20, 0.22%) and weakness (n = 22, 0.26%) were related clinical features. Other involved organs included the eyes (n = 8, 0.16%), nose (n = 7, 0.14%), mouth (n = 6, 0.12%), larynx (n = 1, 0.02%), and scrotum (n = 1, 0.02%) (Table 3).

### Symptoms related to MB and PB leprosy

**Fig 2:**
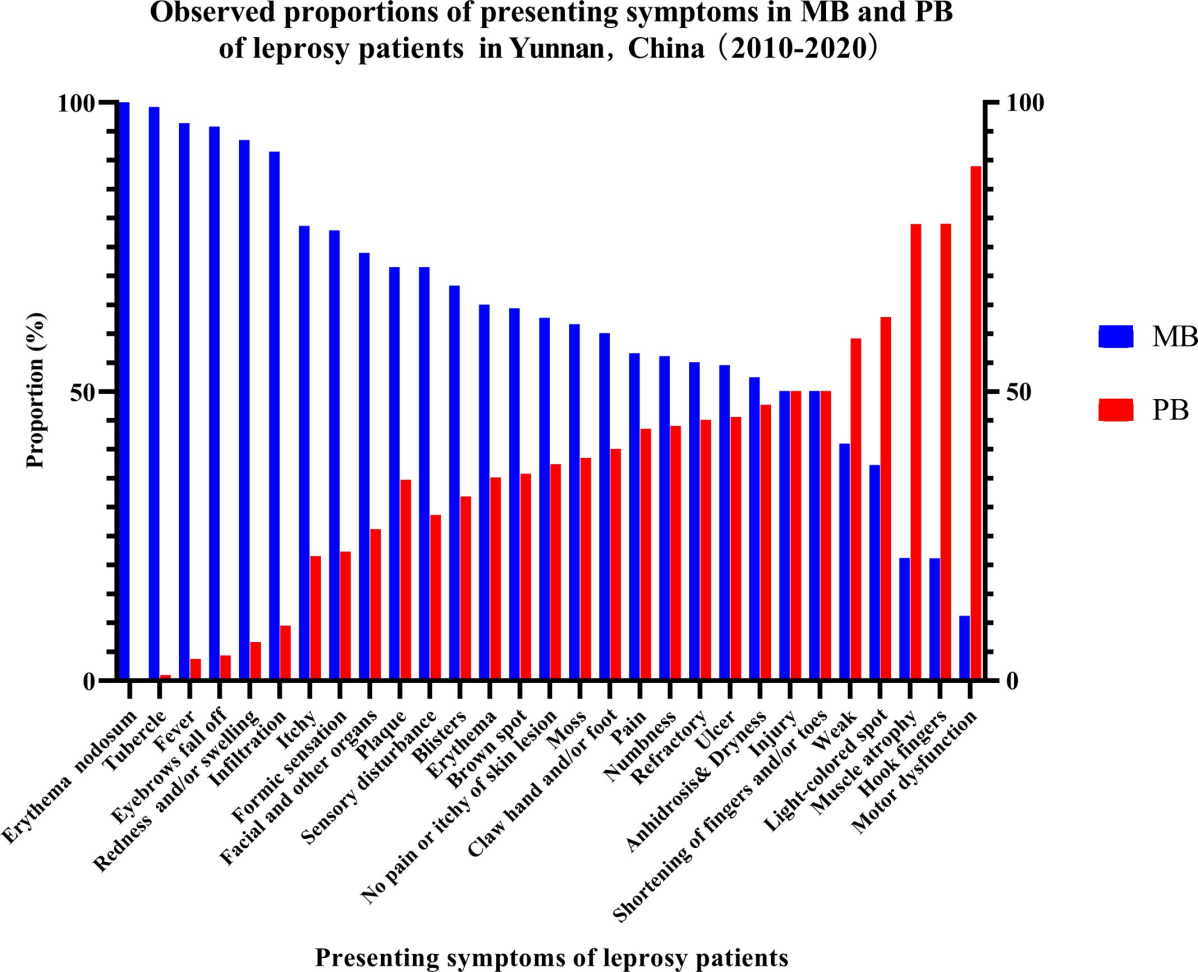
Observed Proportions of Presenting Symptoms in MB and PB Leprosy Patients.

The distributions of symptoms in MB and PB patients are shown in Fig 2. Similar proportions of clinical symptoms were found in MB and PB patients: injury [MB *vs*. PB, 50.00% (7/14) *vs*. 50.00% (7/14)], refractory illness [MB *vs*. PB, 55.00% (11/20) *vs*. 45.00% (9/20)] and weakness [MB *vs*. PB, 40.91% (9/22) *vs*. 59.09% (13/22)] (Table 4). With regard to symptoms of skin appendages, no significant differences in anhidrosis and dryness (MB *vs*. PB, 52.38% (99/189) *vs*. 47.67% (90/189)] were present between MB and PB patients, while eyebrow hair loss [MB *vs*. PB, 95.72% (447/467) *vs*. 4.28% (20/467)] was predominant in MB patients (Table 4). Among those reporting skin lesion-related symptoms, light-colored spots mainly occurred in PB patients [MB *vs*. PB, 37.21% (48/129) *vs*. 62.79% (81/129), while other skin lesions, including erythema (521/802, 64.69%), tubercles (438/442, 99.10%), blisters (73/107, 68.22%), papules (165/231, 71.43%), and infiltration (127/139, 91.37%) mainly occurred in MB patients (Table 4). With regard to nerve symptoms, the proportions of numbness [MB *vs*. PB, 56.34% (464/828) *vs*. 43.66% (364/828)] and pain [MB *vs*. PB, 56.52% (52/92) *vs*. 43.48% (40/92)] were similar between the MB and PB patient groups (Table 4). Painless nor pruritic skin lesions (408/651, 62.67%), formic sensation (77/99, 77.78%), pruritus (44/56, 78.57%) and sensory disturbance (35/49, 71.43%) were predominant in the MB group (Table 4). Symptoms related to leprosy reactions, including redness and/or swelling (240/257, 93.39%), fever (26/27, 96.30%), erythema nodosum (13/13, 100.00%), and facial features (17/23, 73.91%), mainly occurred in the MB group (Table 4). In contrast, some symptoms related to disability, such as finger contracture (82/109, 78.90%), muscle atrophy (56/71, 78.87%), and motor dysfunction (16/18, 88.89%), mainly occurred in the PB group, while ulcer [MB *vs*. PB: 54.46% (55/101) *vs*. 45.54% (46/101)] and shortening of the fingers and toes [MB *vs*. PB: 50.00% (6/12) *vs*. 50.00% (6/12)] occurred at similar rates in the MB and PB groups (Table 4). The odds ratios of symptoms in MB and PB patients are shown in S1 Table.

**Table 4:** Observed Proportions of Presenting Symptoms in MB and PB of Newly Detected Leprosy Cases in Yunnan, China 2010–2020.

Symptoms	Leprosy(n, %)		95%CI		MB(n, %)		95%CI		PB(n, %)		95%CI		P value
Injury	14	0.28%	0.13%	0.43%	7	0.20%	0.05%	0.35%	7	0.45%	0.12%	0.77%	>0.9999
Refractory	20	0.40%	0.23%	0.57%	11	0.32%	0.13%	0.51%	9	0.57%	0.20%	0.95%	0.7524
Weak	22	0.44%	0.26%	0.62%	9	0.26%	0.09%	0.43%	13	0.83%	0.38%	1.27%	0.366
Eyebrows fall off	467	9.34%	8.53%	10.15%	447	12.99%	11.86%	14.11%	20	1.27%	0.72%	1.83%	<0.0001*
Anhidrosis& dryness	189	3.78%	3.25%	4.31%	99	2.88%	2.32%	3.43%	90	5.73%	4.58%	6.87%	0.4106
Erythema	802	16.04%	15.02%	17.06%	521	15.14%	13.94%	16.33%	281	17.88%	15.98%	19.77%	<0.0001*
Tubercle	442	8.84%	8.05%	9.63%	438	12.73%	11.61%	13.84%	4	0.25%	0.01%	0.50%	<0.0001*
Plaque	231	4.62%	4.04%	5.20%	165	4.79%	4.08%	5.51%	80	5.09%	4.00%	6.18%	<0.0001*
Infiltration	139	2.78%	2.32%	3.24%	127	3.69%	3.06%	4.32%	12	0.76%	0.33%	1.19%	<0.0001**
Light-colored spot	129	2.58%	2.14%	3.02%	48	1.39%	1.00%	1.79%	81	5.15%	4.06%	6.25%	<0.0001*
Blisters	107	2.14%	1.74%	2.54%	73	2.12%	1.64%	2.60%	34	2.16%	1.44%	2.88%	<0.0001*
Brown spot	14	0.28%	0.13%	0.43%	9	0.26%	0.09%	0.43%	5	0.32%	0.04%	0.60%	0.2568
Moss	13	0.26%	0.12%	0.40%	8	0.23%	0.07%	0.39%	5	0.32%	0.04%	0.60%	0.4338
Numbness	828	16.56%	15.53%	17.59%	464	13.48%	12.34%	14.62%	364	23.16%	21.07%	25.24%	<0.0001*
Painless nor pruritus of skin lesion	651	13.02%	12.09%	13.95%	408	11.85%	10.77%	12.93%	243	15.46%	13.67%	17.25%	<0.0001*
Formic sensation	99	1.98%	1.59%	2.37%	77	2.24%	1.74%	2.73%	22	1.40%	0.82%	1.98%	<0.0001*
Pain	92	1.84%	1.47%	2.21%	52	1.51%	1.10%	1.92%	40	2.54%	1.77%	3.32%	0.1046
Pruritus	56	1.12%	0.83%	1.41%	44	1.28%	0.90%	1.65%	12	0.76%	0.33%	1.19%	<0.0001*
Sensory disturbance	49	0.98%	0.71%	1.25%	35	1.02%	0.68%	1.35%	14	0.89%	0.43%	1.36%	<0.0001*
Redness and/or swelling	257	5.14%	4.53%	5.75%	240	6.97%	6.12%	7.82%	17	1.08%	0.57%	1.59%	<0.0001*
Fever	27	0.54%	0.34%	0.74%	26	0.76%	0.47%	1.04%	1	0.06%	-0.06%	0.19%	<0.0001*
Erythema nodosum	13	0.26%	0.12%	0.40%	13	0.38%	0.17%	0.58%	0	0.00%	0.00%	0.00%	<0.0001*
Finger contracture	109	2.18%	1.78%	2.58%	23	0.67%	0.40%	0.94%	86	5.47%	4.35%	6.59%	<0.0001*
Ulcer	101	2.02%	1.63%	2.41%	55	1.60%	1.18%	2.02%	46	2.93%	2.09%	3.76%	0.2602
Muscle atrophy*	71	1.42%	1.09%	1.75%	15	0.44%	0.22%	0.66%	56	3.56%	2.65%	4.48%	<0.0001*
Shortening of fingers and/or toes	12	0.24%	0.10%	0.38%	6	0.17%	0.03%	0.31%	6	0.38%	0.08%	0.69%	>0.9999
Motor dysfunction	18	0.36%	0.19%	0.53%	2	0.06%	-0.02%	0.14%	16	1.02%	0.52%	1.51%	<0.0001*
Claw hand and/or foot	5	0.10%	0.01%	0.19%	3	0.09%	-0.01%	0.19%	2	0.13%	-0.05%	0.30%	>0.9999
Facial features and others	23	0.46%	0.27%	0.65%	17	0.49%	0.26%	0.73%	6	0.38%	0.08%	0.69%	0.0028*
Eyelids Incomplete closure	7	0.14%	0.04%	0.24%	4	0.12%	0.00%	0.23%	3	0.19%	-0.02%	0.41%	>0.9999
Red eyes	1	0.02%	-0.02%	0.06%	1	0.03%	-0.03%	0.09%	0	0.00%	0.00%	0.00%	>0.9999
Angle of mouth askew	6	0.12%	0.02%	0.22%	4	0.12%	0.00%	0.23%	2	0.13%	-0.05%	0.30%	0.5671
Nasal symptoms	7	0.14%	0.04%	0.24%	6	0.17%	0.03%	0.31%	1	0.06%	-0.06%	0.19%	0.0291*
Larynx	1	0.02%	-0.02%	0.06%	1	0.03%	-0.03%	0.09%	0	0.00%	0.00%	0.00%	>0.9999
Scrotum	1	0.02%	-0.02%	0.06%	1	0.03%	-0.03%	0.09%	0	0.00%	0.00%	0.00%	>0.9999

MB: multibacillary, PB: paucibacillary.

*P<0.05.

### Symptoms related to early and delayed diagnoses

**Fig 3:**
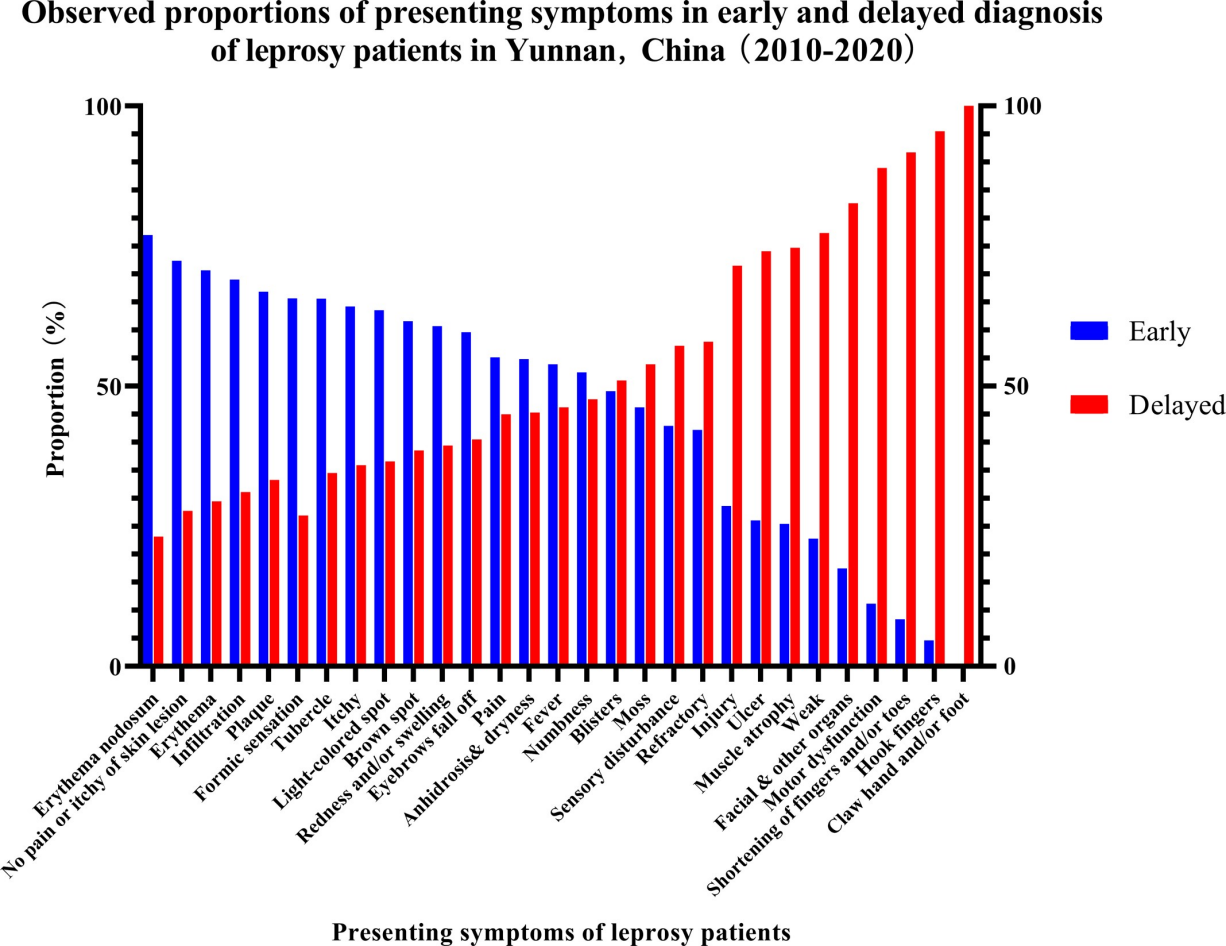
Observed Proportions of Presenting Symptoms in Leprosy Patients with Early and Delayed Diagnosis.

As 56 records were missing information about the stage of leprosy, 2069 newly detected leprosy patients were included in the analysis of symptoms related to early or delayed diagnosis. In total, 1249 (58.78%) and 820 (38.59%) patients were divided into early and delayed diagnosis groups, respectively. The proportions of symptoms in the early and delayed diagnosis groups are shown in Fig 3. Injury (10/14, 71.43%), refractory illness (11/19, 57.89%) and weakness (17/22, 77.27%), the symptoms of clinical features, were mainly present in the delayed diagnosis group. Despite the substantial proportion of patients with early diagnosis, few newly detected leprosy patients in the early diagnosis group had recorded clinical features such as injury (4/14, 28.57%), refractory illness (3/9, 33.33%), or weakness (5/22, 22.73%) (Table 5). With regard to symptoms of skin appendages, no significant differences in eyebrow hair loss (59.59% *vs*. 41.32%) or anhidrosis and dryness [59.59% (261/438) *vs*. 40.41% (177/438)] was observed between the early and delayed diagnosis groups (Table 5). Among those reporting skin lesion-related symptoms, erythema (555/786, 70.61%), tubercles (274/418, 65.55%), infiltration (91/132, 68.94%), and light-colored spots (80/126, 63.49%) mainly occurred in the early diagnosis group (Table 5). With regard to nerve symptoms, the rates of numbness [52.40% (425/811) *vs*. 47.60% (386/811)] and pain [55.06% (49/89) *vs*. 44.94% (40/89)] were similar between the early and delayed diagnosis groups (Table 5). Painless nor pruritic skin lesions (460/636, 72.33%), formic sensation (61/93, 65.59%) and pruritus (34/53, 64.15%) were predominant in the early diagnosis group (Table 5). Symptoms related to leprosy reactions mainly occurred in the early diagnosis group: redness and/or swelling (151/249, 60.64%), fever (14/26, 53.85%), and erythema nodosum (10/13, 76.92%) (Table 5). In contrast, symptoms related to disability, such as finger contracture (104/109, 95.41%), ulcers (74/100, 74.00%), muscle atrophy (53/71, 74.65%), motor dysfunction (16/18, 88.89%), shortening of the fingers and toes (11/12, 91.67%), claw hands and/or foot deformity (5/5, 100.00%), and facial features (19/23, 82.61%), mainly occurred in the delayed diagnosis group (Table 5). The odds ratios of symptoms in the early diagnosis group are shown in S2 Table.

**Table 5:** Observed Proportions of Early Diagnosis and Delayed Diagnosis Associated with Presenting Symptoms in Newly Detected Leprosy Cases in Yunnan, China, 2010–2020.

Symptoms	Leprosy (n,%)		95%CI		Early diagnosis(n, %)		95%CI		Delayed diagnosis(n, %)		95%CI		P value
Injury	14	0.29%	0.14%	0.44%	4	0.14%	0.00%	0.28%	10	0.51%	0.19%	0.82%	0.057
Refractory	19	0.39%	0.22%	0.57%	8	0.28%	0.09%	0.47%	11	0.56%	0.23%	0.88%	0.5171
Weak	22	0.45%	0.26%	0.64%	5	0.17%	0.02%	0.33%	17	0.86%	0.45%	1.27%	0.0007*
Eyebrows fall off	438	9.02%	8.21%	9.82%	261	9.07%	8.02%	10.11%	177	8.98%	7.69%	10.20%	<0.0001*
Anhidrosis& dryness	188	3.87%	3.33%	4.41%	103	3.58%	2.90%	4.26%	85	4.31%	3.40%	5.19%	0.0794
Erythema	786	16.18%	15.14%	17.22%	555	19.28%	17.84%	20.72%	231	11.71%	10.26%	13.09%	<0.0001*
Tubercle	418	8.60%	7.82%	9.39%	274	9.52%	8.45%	10.59%	144	7.30%	6.13%	8.42%	<0.0001*
Plaque	226	4.65%	4.06%	5.24%	151	5.24%	4.43%	6.06%	75	3.80%	2.95%	4.63%	<0.0001*
Infiltration	132	2.72%	2.26%	3.17%	91	3.16%	2.52%	3.80%	41	2.08%	1.44%	2.70%	<0.0001*
Light-colored spot	126	2.59%	2.15%	3.04%	80	2.78%	2.18%	3.38%	46	2.33%	1.66%	2.99%	<0.0001*
Blisters	106	2.18%	1.77%	2.59%	52	1.81%	1.32%	2.29%	54	2.74%	2.01%	3.45%	0.8908
Brown spot	13	0.27%	0.12%	0.41%	8	0.28%	0.09%	0.47%	5	0.25%	0.03%	0.47%	0.4338
Moss	13	0.27%	0.12%	0.41%	6	0.21%	0.04%	0.37%	7	0.35%	0.09%	0.62%	>0.9999
Numbness	811	16.69%	15.65%	17.74%	425	14.76%	13.47%	16.06%	386	19.57%	17.76%	21.25%	0.0591
Painless nor pruritus skin lesions	636	13.09%	12.14%	14.04%	460	15.98%	14.64%	17.32%	176	8.92%	7.64%	10.15%	<0.0001*
Formic sensation	93	1.91%	1.53%	2.30%	61	2.12%	1.59%	2.64%	32	1.62%	1.06%	2.17%	<0.0001*
Pain	89	1.83%	1.45%	2.21%	49	1.70%	1.23%	2.17%	40	2.03%	1.40%	2.64%	0.2303
Pruritus	53	1.09%	0.80%	1.38%	34	1.18%	0.79%	1.58%	19	0.96%	0.53%	1.39%	0.0063*
Sensory disturbance	49	1.01%	0.73%	1.29%	21	0.73%	0.42%	1.04%	28	1.42%	0.89%	1.94%	0.2253
Redness and/or swelling	249	5.13%	4.51%	5.75%	151	5.24%	4.43%	6.06%	98	4.97%	4.00%	5.91%	<0.0001*
Fever	26	0.54%	0.33%	0.74%	14	0.49%	0.23%	0.74%	12	0.61%	0.26%	0.95%	0.7819
Erythema nodosum	13	0.27%	0.12%	0.41%	10	0.35%	0.13%	0.56%	3	0.15%	-0.02%	0.32%	0.0169*
Finger contracture	109	2.24%	1.83%	2.66%	5	0.17%	0.02%	0.33%	104	5.27%	4.27%	6.24%	<0.0001*
Ulcer	100	2.06%	1.66%	2.46%	26	0.90%	0.56%	1.25%	74	3.75%	2.90%	4.58%	<0.0001*
Muscle atrophy*	71	1.46%	1.12%	1.80%	18	0.63%	0.34%	0.91%	53	2.69%	1.97%	3.39%	<0.0001*
Motor dysfunction	18	0.37%	0.20%	0.54%	2	0.07%	-0.03%	0.17%	16	0.81%	0.41%	1.20%	<0.0001*
Shortening of fingers and/or toes	12	0.25%	0.11%	0.39%	1	0.03%	-0.03%	0.10%	11	0.56%	0.23%	0.88%	0.0001*
Claw hand and/or foot	5	0.10%	0.01%	0.19%	0	0.00%	0.00%	0.00%	5	0.25%	0.03%	0.47%	0.0079*
Facial features and others	23	0.47%	0.28%	0.67%	4	0.14%	0.00%	0.28%	19	0.96%	0.53%	1.39%	<0.0001*
Eyelids Incomplete closure	7	0.14%	0.04%	0.25%	0	0.00%	0.00%	0.00%	7	0.35%	0.09%	0.62%	0.0006*
Red eyes	1	0.02%	-0.02%	0.06%	0	0.00%	0.00%	0.00%	1	0.05%	-0.05%	0.15%	>0.9999
Angle of mouth askew	6	0.12%	0.02%	0.22%	1	0.03%	-0.03%	0.10%	5	0.25%	0.03%	0.47%	0.0801
Nasal symptoms	7	0.14%	0.04%	0.25%	2	0.07%	-0.03%	0.17%	5	0.25%	0.03%	0.47%	0.2861
Larynx	1	0.02%	-0.02%	0.06%	0	0.00%	0.00%	0.00%	1	0.05%	-0.05%	0.15%	>0.9999
Scrotum	1	0.02%	-0.02%	0.06%	1	0.03%	-0.03%	0.10%	0	0.00%	0.00%	0.00%	>0.9999

*P<0.05.

### Initial symptoms at symptom onset and only symptoms at diagnosis

A total of 2069 and 2125 newly detected leprosy patients were included in the analyses of initial symptoms and only symptoms related to physical disability and diagnosis interval, respectively. The initial symptoms at symptom onset and the only symptoms at diagnosis were divided into skin, skin and nerve, and nerve symptoms (Table 6). The proportions of skin, skin and nerve, and nerve symptoms as initial symptoms were 33.25%, 44.95%, and 21.80% and as only symptoms were 28.66%, 57.81%, and 13.53%, respectively. Regarding initial symptoms, the proportions of patients with physical disability (G1D + G2D) were 28.92%, 30.32% and 57.65% for skin, skin and nerve, and nerve symptoms, respectively. Regarding only symptoms, the proportions were 25.97%, 33.78% and 65.36%, respectively. Similarly, regarding skin, skin and nerve, and nerve symptoms as initial symptoms, the proportions of patients with a diagnosis interval of more than 5 years were 8.72%, 5.70%, and 15.97%, respectively. For these symptoms as the only symptoms, the proportions were 7.25%, 7.78%, and 17.50%, respectively (Table 6). In addition, for skin, skin and nerve, and nerve symptoms as the initial symptoms, the means±standard deviations (SDs) of the diagnosis intervals were 28.77±31.55, 25.60±26.20, and 38.88±46.02, respectively. For these symptoms as the only symptoms, the means±SDs of the diagnosis intervals were 27.52±28.89, 28.07±30.66, and 40.35±49.36, respectively (Table 6).

**Table 6:** Initial Symptoms at Symptom Onset and Only Symptoms at Diagnosis in Newly Detected Leprosy Cases in Yunnan, China, 2010–2020.

Presenting symptoms		First symptoms(n, %)						Only Symptoms(n, %)					
		Skin		Skin & nerve		Nerve		Skin		Skin & nerve		Nerve	
**Physical disability**	Total	688	33.25%	930	44.95%	451	21.80%	593	28.66%	1196	57.81%	280	13.53%
	G0D	489	71.08%	648	69.68%	191	42.35%	439	74.03%	792	66.22%	97	34.64%
	G1D	111	16.13%	127	13.66%	88	19.51%	93	15.68%	176	14.72%	57	20.36%
	G2D	88	12.79%	155	16.67%	172	38.14%	61	10.29%	228	19.06%	126	45.00%
	G1D+G2D	199	28.92%	282	30.32%	260	57.65%	154	25.97%	404	33.78%	183	65.36%
**Diagnosis interval (years)**	Total	708	33.32%	953	44.85%	464	21.83%	603	28.38%	1238	58.26%	284	13.36%
	≤2 y	492	69.49%	700	73.45%	245	52.80%	432	71.64%	856	69.14%	149	52.46%
	2–5 y	156	22.03%	200	20.99%	146	31.47%	129	21.39%	287	23.18%	86	30.28%
	5–10 y	43	6.07%	36	3.78%	45	9.70%	29	4.81%	68	5.49%	27	9.51%
	≥10 y	17	2.40%	17	1.78%	28	6.03%	13	2.16%	27	2.18%	22	7.75%
	≥5 y	60	8.47%	53	5.56%	73	15.73%	42	6.97%	95	7.67%	49	17.25%
**Diagnosis interval (months)**	Median (IQR)	20 (17–26)		20 (17–25)		23 (17–39)		20 (17–25)		20 (17–27)		23 (17–40)	
	Min-Max	1–341		1–360		1–364		1–273		1–361		1–364	
	Mean±SD	28.77±31.55		25.60±26.20		38.88±46.02		27.52±28.89		28.07±30.66		40.35±49.36	
	95%CI of Mean	26.44–31.10		23.97–27.26		34.68–43.08		25.21–29.83		26.36–29.78		34.59–46.12	

IQR:interquartile range.

## Discussion

Understanding the symptoms associated with leprosy, the subtypes of leprosy, and the diagnostic interval will help guide early diagnosis initiatives. In this retrospective analysis, we described the main complaints in newly detected leprosy patients over the past decade in Yunnan, China, a region with a high burden of leprosy, and revealed the common symptoms of leprosy and the symptoms associated with different subtypes of leprosy and different diagnostic intervals using data from a population-based cohort of patients with incident leprosy.

Information about presenting symptoms in the patient population was obtained via symptom self-reports and record-based symptom information extraction. To collect data on self-reported symptoms, information about presenting symptoms can be directly elicited from patients through semistructured interviews or questionnaires [9]. Alternatively, for record-based symptom information collection, information on presenting symptoms can be recorded during healthcare encounters (e.g., with a primary care physician) and captured as part of the patient’s health record [9]. In this study, we used the latter approach, as self-reported symptoms captured retrospectively (after diagnosis) would have a high risk of bias [9].

Presenting symptoms have been reported in numerous studies and included weakness [10], anhidrosis and dryness [10], eyebrow hair loss [11], eye closure weakness [12], a collapsed nose [13], edema [14], erythema nodosum [15], numbness [16], loss of sensation [10], pain [17], pruritus [18], muscle atrophy [19], and claw hand deformity [20,21]. Initially, the small sensory and autonomic nerve fibers in the skin are damaged, causing local loss of hair, an inability to sweat and difficulty detecting temperature and touch sensations. Damage to peripheral nerves can lead to widespread skin dryness, loss of sensation, and weakness or paralysis of the muscles in areas of the body supplied by the affected nerve. Eyes, hands, and feet with loss of sensation, paralysis or dryness have an increased risk of injury. Dry skin can lead to cracks. If cracks, injuries, and ulcerations are not cared for and healed, they can become infected, leading to further injury and destruction, resulting in visible damage and destruction of the eyes, hands, and feet. These are easily observed impairments; these injuries, along with paralysis, are obvious and considered grade 2 disability in leprosy [10].

A symptom signature denotes the nature and relative frequency of symptoms (or symptom combinations) at presentation to the medical facility by patients later diagnosed with leprosy or with a particular subtype or stage of leprosy. As leprosy mainly affects the skin and peripheral nervous system, skin symptoms and nerve symptoms were predominant. Numbness (nerve symptoms), erythema (skin lesions), painless nor pruritic skin lesions (nerve symptoms), eyebrow hair loss (skin appendage), and tubercles (skin lesion) were common symptoms of leprosy. Despite the low proportions, nerve (formic sensation, pain, and pruritus) and disability (finger contracture, muscle atrophy, and motor dysfunction) symptoms, which have been seldom mentioned as physical symptoms of leprosy previously, were obvious in this study.

Among the newly detected cases of leprosy with symptom signatures in this study, the majority of cases were MB cases, while minority were PB cases. Describing only the symptoms related to leprosy would lead to bias in the presenting symptoms; thus, we analyzed the symptoms related to the subtypes of leprosy in detail. Light-colored spots and pale-white spots mainly occurred in PB cases, while other skin symptoms, including erythema, eyebrows hair loss, tubercles, blisters, plaques, infiltration, and papules, mainly occurred in MB cases. Symptoms related to leprosy reactions and facial features mainly occurred in MB cases, while symptoms related to physical disability, such as finger contracture, muscle atrophy, and motor dysfunction, mainly occurred in the PB group. In addition, painless nor pruritic skin lesions, formic sensation, pruritus, and sensory disturbance were predominant in the MB group. The diverse clinical manifestations are strongly correlated with the host’s immune response to *M*. *leprae* [22]. Symptom signatures can be described as “narrow”, when most patients present with a particular symptom, or “broad”, when patients present with a wide range of symptoms [9]; eyebrow hair loss, tubercles, papules, and symptoms related to leprosy reactions, such as redness and/or swelling, fever, and erythema nodosum, which all had proportions higher than 90% in MB cases, were considered narrow signatures of MB leprosy. In this study, we also found that finger contracture, muscle atrophy, and motor dysfunction were potential symptoms of PB leprosy.

Early diagnosis is an important aspect of leprosy control strategies, as early case detection, regular and complete MDT, and early detection of impairment and disability have played a pivotal role in reducing the disease and disability burdens in the community [2]. Awareness of presenting symptoms will improve the level of leprosy awareness in the community and the capacity of the health system to recognize leprosy early, thus influencing the length of the interval from symptom onset to presentation (the patient interval) and from initial presentation to specialist referral (the primary care interval) [5]. We described the symptoms related to the different stages of diagnosis. All the skin and leprosy reactions symptoms and some of the nerve symptoms, including painless nor pruritic skin lesions, formic sensation, and pruritus, mainly existed in the early diagnosis group, while all symptoms related to clinical features, facial features and physical disability mainly occurred in the delayed diagnosis group. Finger contracture, shortening of the fingers and toes, and claw hand and/or foot deformity were regarded as narrow signatures and associated with delayed diagnosis of leprosy. It is worth noting that painless nor pruritic skin lesions, formic sensation and pruritus occurred in not only the MB group but also the early diagnosis group, while symptoms related to physical disability, such as finger contracture, muscle atrophy, and motor dysfunction, were associated with not only PB but also delayed diagnosis, which implies that diagnosis of PB leprosy, which usually presents as physical disability even in the early stage of disease onset, is a critical problem that needs further research.

Leprosy neuropathy is considered the most common peripheral neuropathy with an infectious etiology worldwide and a public health problem [23]. Diagnosing leprosy in the absence of typical dermatological features is challenging, and a lack of features frequently causes a delay in diagnosis [24]. Primary neural leprosy (PNL), also known as pure neural or neuritic leprosy, was initially described in the Indian classification in 1955 [25]. Since then, it has been a challenge to clinically diagnose, as no skin lesions occur and slit skin smear bacilloscopy is negative. In a previous study, 4–8% (up to 18% in some Indian case series) of patients with leprosy may present with PNL, characterized by evidence of nerve deficit or thickening, with or without tenderness in the absence of skin involvement [26]. According to another Indian study, on average, PNL accounts for 5–17.7% of all leprosy cases [27]. In this study, 21.80% and 13.53% of the newly detected leprosy patients presented with nerve symptoms as the first symptoms and the only symptoms, respectively, consistent with previous studies. Despite having the lowest patient proportion, the nerve symptom group had the highest proportion of physical disability and the longest diagnosis interval compared with the skin and skin and nerve groups. These results imply that we should increase awareness of nerve symptoms. Individuals with nerve symptoms should be considered the new target population, neurology outpatients should undergo leprosy screening, and focused training should be provided for specialized neurology medical staff to enhance the capacity of health systems to recognize leprosy early.

In summary, this study provides a detailed description of the symptom signatures of leprosy, including MB/PB leprosy, leprosy with an early/delayed diagnosis, leprosy for which the first/only symptoms are physical disability and leprosy diagnosed at different intervals, among newly detected leprosy patients. Numbness, erythema, painless nor pruritic skin lesions, eyebrow hair loss, and tubercles were common symptoms of leprosy. Despite their low proportions, formic sensation, pain, pruritus, finger contracture, muscle atrophy, and motor dysfunction were reported during the diagnosis of leprosy. In detail, finger contracture, muscle atrophy, and motor dysfunction mainly occurred in the PB group. These symptoms, along with other symptoms related to disability, were predominantly in the delayed diagnosis group. This implies that leprosy diagnosis, especially PB leprosy diagnosis, is still a challenge. As nerve symptoms were related to physical disability and a longer diagnosis interval, increasing awareness of nerve symptoms would be helpful for preventing physical disability and promoting the early detection of leprosy cases.

To our knowledge, this is the largest study to date to examine the symptoms of newly detected leprosy cases before diagnosis. Our findings are based on a large cohort of newly detected leprosy patients and self-reported data on symptoms, WHO classification, diagnosis interval and physical disability. The study participants were largely representative of leprosy patients nationwide, with some underrepresentation of reversal reaction and erythema nodosum leprosum.

This study has some limitations. First, as there is no standard assessment tool for risk of bias in observational nonrandomized studies, we could not evaluate the risk of bias. Second, because the disease exhibits different dermatologic and neurologic manifestations within a wide clinical spectrum, many diseases should be considered in the differential diagnosis [1]. We described only the symptoms relevant to leprosy and did not describe the symptoms used to form the differential diagnosis. Despite suspected symptoms, a leprosy diagnosis still needs to be confirmed with a special medical examination performed by trained medical staff according to the criteria of leprosy diagnosis. Third, the clinical features of the disease depend on bacterial proliferation, the immunologic response of the host to *M*. *leprae*, and peripheral neural involvement. According to the clinical features, leprosy can be divided into indeterminate leprosy, TT, BT, BB, BL, LL, type I leprosy reaction (reversal reaction), type II leprosy reaction, and some rare clinical forms, such as pure neural-type leprosy, histoid leprosy, localized lepromatous or borderline disease, and Lucio leprosy (lepra bonita) (erythema nodosum leprosum reaction) [1]. The number of leprosy patients enrolled in this study was limited; therefore, it was difficult to cover every subtype of leprosy. Further investigations in larger populations will increase confidence in the diagnostic and discriminatory value of the suspected symptoms.

## Conclusions

In conclusion, the diagnosis of leprosy is still challenging because leprosy is one of “the great imitators” and a disease with “many faces” [1]. Expanding knowledge about the presenting symptoms will be helpful in the detection new leprosy cases. Elucidating suspected symptoms of leprosy would improve the clinical diagnosis rate and promote early diagnosis, thereby preventing physical disability in leprosy patients.

## Supporting information

S1 Table Odds ratios of MB of leprosy disease by presenting symptoms. (XLSX)

S2 Table Odds ratios of early diagnosis of leprosy disease by presenting symptoms. (XLSX)

## References

[1] Kundakci N, Erdem C. Leprosy: a great imitator. Clin Dermatol. 2019;37: 200–212. doi: 10.1016/j.clindermatol.2019.01.002 31178103

[2] Naaz F, Mohanty PS, Bansal AK, Kumar D, Gupta UD. Challenges beyond elimination in leprosy. Int J Mycobacteriol. 2017;6: 222–228. doi: 10.4103/ijmy.ijmy_70_17 28776519

[3] Global leprosy update, 2016: accelerating reduction of disease burden. Wkly Epidemiol Rec. 2017;92: 501–519. 28861986

[4] Gómez L, Rivera A, Vidal Y, Bilbao J, Kasang C, Parisi S, et al. Factors associated with the delay of diagnosis of leprosy in north-eastern Colombia: a quantitative analysis. Trop Med Int Health. 2018;23: 193–198. doi: 10.1111/tmi.13023 29230912

[5] Da Silva Souza C, Bacha JT. Delayed diagnosis of leprosy and the potential role of educational activities in Brazil. Lepr Rev. 2003;74: 249–258. 14577470

[6] Leon KE, Jacob JT, Franco-Paredes C, Kozarsky PE, Wu HM, Fairley JK. Delayed diagnosis, leprosy reactions, and nerve injury among individuals with hansen’s disease seen at a United States clinic. Open Forum Infect Dis. 2016;3: ofw063. doi: 10.1093/ofid/ofw063 27186586 PMC4866574

[7] Ridley DS, Jopling WH. Classification of leprosy according to immunity. A five-group system. Int J Lepr Other Mycobact Dis. 1966;34: 255–273. 5950347

[8] WHO. World health organization expert committee on leprosy: Seventh report. Technical Report Series 847. Geneva: WHO; 1998.

[9] Koo MM, Hamilton W, Walter FM, Rubin GP, Lyratzopoulos G. Symptom signatures and diagnostic timeliness in cancer patients: a review of current evidence. Neoplasia. 2018;20: 165–174. doi: 10.1016/j.neo.2017.11.005 29253839 PMC5735300

[10] Lockwood D, Colston MJ, Fine PE, Lucas S, Rose SP, McDougall AC. Prevention of disabilities and rehabilitation. Lepr Rev. 2002;73: S35–S43.

[11] Ferreira RC, Gonçalves TX, Soares A, Carvalho LRA, Campos FL, Ribeiro MTF, et al. Dependence on others for oral hygiene and its association with hand deformities and functional impairment in elders with a history of leprosy. Gerodontology. 2018. doi: 10.1111/ger.12346 29781555

[12] Reinar LM, Forsetlund L, Lehman LF, Brurberg KG. Interventions for ulceration and other skin changes caused by nerve damage in leprosy. Cochrane Database Syst Rev. 2019;7: CD012235. doi: 10.1002/14651858.CD012235.pub2 31425632 PMC6699662

[13] Husain S. Reconstruction of moderately depressed nose in leprosy (a long-term follow-up). Indian J Lepr. 2013;85: 115–121. 24724233

[14] Chandan N, Ashack KA, Hill C. Ulcers and edema in a patient with leprosy. Int J Dermatol. 2020;59: 1073–1075. doi: 10.1111/ijd.14845 32185795

[15] Narang T, Ashraf R, Kaushik A, Dogra S. Apremilast in multibacillary leprosy patients with chronic and recurrent erythema nodosum leprosum: a prospective single-centre pilot study. J Eur Acad Dermatol Venereol. 2021. doi: 10.1111/jdv.17585 34365679

[16] Hanewinckel R, van Oijen M, Ikram MA, van Doorn PA. The epidemiology and risk factors of chronic polyneuropathy. Eur J Epidemiol. 2016;31: 5–20. doi: 10.1007/s10654-015-0094-6 26700499 PMC4756033

[17] Raicher I, Stump PR, Baccarelli R, Marciano LH, Ura S, Virmond MC, et al. Neuropathic pain in leprosy. Clin Dermatol. 2016;34: 59–65. doi: 10.1016/j.clindermatol.2015.10.012 26773624

[18] Noormohammadpour P, Kamyab-Hesari K, Razavi Z, Dolatyabi N, Taghizadeh Fazli J, Daneshpazhooh M, et al. An unusual case of multibacillary leprosy mimicking prurigo nodularis. Clin Case Rep. 2020;8: 1234–1237. doi: 10.1002/ccr3.2897 32695365 PMC7364099

[19] Shrestha BK, Ranabhat K, Pant R, Sapkota S, Shrestha S. Neuritic leprosy; an intriguing re-visit to a forbidden ailment. Kathmandu Univ Med J (KUMJ). 2019;17: 73–76. 31734684

[20] Miyashiro D, Cardona C, Valente NYS, Avancini J, Benard G, Trindade MAB. Ulcers in leprosy patients, an unrecognized clinical manifestation: a report of 8 cases. BMC Infect Dis. 2019;19: 1013. doi: 10.1186/s12879-019-4639-2 31783808 PMC6884743

[21] Riyaz N, Sehgal VN. Leprosy: trophic skin ulcers. Skinmed. 2017;15: 45–51. 28270310

[22] Froes LAR Jr., Trindade MAB, Sotto MN. Immunology of leprosy. Int Rev Immunol. 2020: 1–21. doi: 10.1080/08830185.2020.1851370 33241709

[23] Santos DFD, Mendonça MR, Antunes DE, Sabino EFP, Pereira RC, Goulart LR, et al. Revisiting primary neural leprosy: clinical, serological, molecular, and neurophysiological aspects. PLoS Negl Trop Dis. 2017;11: e0006086. doi: 10.1371/journal.pntd.0006086 29176796 PMC5720806

[24] Ooi W, Saini S. Diagnostic pitfalls in an atypical case of primary neuritic leprosy. Am J Trop Med Hyg. 2021;105: 490–493. 10.4269/ajtmh.20-1130 PMC8437164 34152999

[25] Indian Association of Leprologists. Classification of leprosy adopted at all India leprosy workers conference. Lepr India. 1955;27: 93.

[26] Wilder-Smith E. Diagnosis of pure neuritic leprosy. Neurol J Southeast Asia. 2002;7: 61–63.

[27] Girdhar BK. Neuritic leprosy. Indian J Lepr. 1996;68: 35–42. 8727112

